# Supplementary material for: From sequence to enzyme mechanism using multi-label machine learning
Source: BMC Bioinformatics. 2014 May 19;15:150. doi: 10.1186/1471-2105-15-150 (PMC4229970; doi:10.1186/1471-2105-15-150)
Supplement: Additional file 2 — Java code of ml2db. Additional file ml2db_code.tar.gz contains the Java source code to run the multi-label machine learning experiments and save the results to database. The code’s Javadoc is included. [file 1471-2105-15-150-S2.zip › additional file 2/ml2db/ml2db/doc/mulan/data/LabelsPair.html]

LabelsPair


JavaScript is disabled on your browser.


- Overview
- Package
- Class
- Use
- Tree
- Deprecated
- Index
- Help

- Prev Class
- Next Class

- Frames
- No Frames

- All Classes

- Summary:
- Nested |
- Field |
- Constr |
- Method

- Detail:
- Field |
- Constr |
- Method


mulan.data

## Class LabelsPair

- java.lang.Object
- - mulan.data.LabelsPair

- All Implemented Interfaces:
  :   java.io.Serializable, java.lang.Comparable

  ---

    

  ```
  public class LabelsPair
  extends java.lang.Object
  implements java.lang.Comparable, java.io.Serializable
  ```

  Class for handling label pairs along with their 'dependence' scores. A pair of labels is represented as an array of two int values.
  The natural order of label pairs is according to natural order of their dependence scores.

  Version:
  :   30.11.2010

  Author:
  :   Lena Chekina (lenat@bgu.ac.il)

  See Also:
  :   Serialized Form

- - ### Constructor Summary

    Constructors

    Constructor and Description

    `LabelsPair(int[] aPair,
    double aScore)`

    Initialize a labels pair using an array of two int values.
  - ### Method Summary

    Methods

    | Modifier and Type | Method and Description |
    | --- | --- |
    | `int` | `compareTo(java.lang.Object otherPair)` |
    | `boolean` | `equals(java.lang.Object o)` |
    | `int[]` | `getPair()` |
    | `java.lang.Double` | `getScore()` |
    | `int` | `hashCode()` |
    | `void` | `setPair(int[] pair)` |
    | `void` | `setScore(java.lang.Double score)` |
    | `java.lang.String` | `toString()` |

    - ### Methods inherited from class java.lang.Object

      `getClass, notify, notifyAll, wait, wait, wait`

- - ### Constructor Detail


    - #### LabelsPair

      ```
      public LabelsPair(int[] aPair,
                double aScore)
      ```

      Initialize a labels pair using an array of two int values.

      Parameters:
      :   `aPair` - - a pair of labels (i.e. an array of length 2)
      :   `aScore` - - a dependence score
  - ### Method Detail


    - #### getPair

      ```
      public int[] getPair()
      ```

      Returns:


    - #### setPair

      ```
      public void setPair(int[] pair)
      ```

      Parameters:
      :   `pair` -


    - #### getScore

      ```
      public java.lang.Double getScore()
      ```

      Returns:


    - #### setScore

      ```
      public void setScore(java.lang.Double score)
      ```

      Parameters:
      :   `score` -


    - #### equals

      ```
      public boolean equals(java.lang.Object o)
      ```

      **Overrides:**
      :   `equals` in class `java.lang.Object`


    - #### compareTo

      ```
      public int compareTo(java.lang.Object otherPair)
      ```

      **Specified by:**
      :   `compareTo` in interface `java.lang.Comparable`


    - #### hashCode

      ```
      public int hashCode()
      ```

      **Overrides:**
      :   `hashCode` in class `java.lang.Object`


    - #### toString

      ```
      public java.lang.String toString()
      ```

      **Overrides:**
      :   `toString` in class `java.lang.Object`

      Returns:
      :   a string representation of a labels pair in the form: "labels pair: [index1, index2]; dependence score; value"


- Overview
- Package
- Class
- Use
- Tree
- Deprecated
- Index
- Help

- Prev Class
- Next Class

- Frames
- No Frames

- All Classes

- Summary:
- Nested |
- Field |
- Constr |
- Method

- Detail:
- Field |
- Constr |
- Method
